# EMPOWER-PD - A physical therapy intervention to empower the individuals with Parkinson’s disease: a study protocol for a feasibility randomized controlled trial

**DOI:** 10.1186/s40814-019-0394-9

**Published:** 2019-01-28

**Authors:** Helena de Oliveira Braga, Elaine Cristina Gregório, Rafaela Simon Myra, Ana Sofia Kauling de Souza, Talita Vitorina Kunh, Jessica Klug, Adriana Coutinho de Azevedo Guimarães, Alessandra Swarowsky

**Affiliations:** Brazilian Parkinson’s Disease Rehabilitation Initiative (BPaRkI). Physical Therapy Postgraduate Program, Physical Therapy Department, Santa Catarina State University (UDESC), Center for Health and Sport Sciences (CEFID), Rua Pascoal Simone, 358, Coqueiros, Florianópolis, Santa Catarina 88080-350 Brazil

**Keywords:** Personal autonomy, Patient participation, Patient-centered care, Qualitative analysis

## Abstract

**Background:**

One of the greatest barriers found by physical therapy treating individuals with Parkinson’s disease (PD) is the adherence to treatment, associated with the lack of motivation to remain active. Therefore, there is a need to change the look given to physical therapy and for the individual, seeking the centralization of the therapy in their preferences. This study aims to present the EMPOWER-PD, a protocol based on individual preferences and its design for feasibility.

**Method:**

A 12-week pilot for a randomized clinical trial will assess the feasibility and preliminary effectiveness of the EMPOWER-PD and make comparisons with conventional physical therapy (CPh). Both protocols consist of mobility and locomotion training, and aim at improving motor and non-motor symptoms through different approaches. The EMPOWER-PD aims to provide a source of motivation and empowerment of health through the self-knowledge of the individual’s abilities and limitations, in a protocol that addresses individual preferences. The CPh is based only on physiotherapist decisions, not addressing the individual’s preferences or motivation/empowerment. The target recruitment will be 24 PD individuals, between stages I and III of Hoehn and Yahr (HY), who will be recruited from Brazilian Parkinson’s disease Rehabilitation Initiative (BPaRkI) with allocation ratio 1:1. A computerized block randomization procedure will be implemented by a blinded researcher with the participants blinded to group assignment. The sessions will be conducted in a group setting, twice a week, during 60 min for 8 weeks, followed by 4 weeks of follow-up. The primary outcomes will be the feasibility data (adherence, recruitment rate, and safety). The secondary outcomes will assess the preliminary efficacy on qualitative assessment about individual’s motivation/empowerment and quantitative motor outcomes (Timed Up and Go and Dynamic Gait Index) and non-motor symptoms (6-min walk test and Fatigue Severity Scale). The recommendation to have 6–12 participants per group will be adopted based on the qualitative analysis to the sample size.

**Discussion:**

This study will provide important insights about the physical therapy approach in PD individuals. The EMPOWER-PD is innovative because (1) it proposes an intervention that includes an individual-centered approach with motor control principles; (2) it aims to provide a source of motivation and empowerment of health; (3) assesses the individual in a global view considering motor and non-motor symptoms, using both, qualitative and quantitative metrics.

**Trial registration:**

RBR-7ZBXQ5

**Electronic supplementary material:**

The online version of this article (10.1186/s40814-019-0394-9) contains supplementary material, which is available to authorized users.

## Background

The global benefits of physical therapy in the domains of International Classification of Functioning, Disability and Health (ICF) [1] in activity, participation, and body structure and function for individuals with Parkinson’s disease (PD) have been clearly established in the literature [2, 3]. However, studies have shown that some individuals present with barriers to the practice of physical therapy, decreasing their motivation towards the treatment and consequently decreasing their adherence [4–6]. Among the strategies suggested both by PD individual’s and by the therapists to engage the individuals in the exercise, a joint work seeking to stimulate self-efficacy and confidence, recognizing and respecting their barriers and individual motivations, were considered of utmost importance [7].

New approaches recommended targeting these challenges include employing the use of individual-centered care [2]. The individual-centered care aims to provide respectful and responsive care to their individual preferences, their needs, and values, ensuring that they guide all clinical decisions [8–10]. The focus is accessing the “person behind the disease,” respecting their preferences and ensuring their inclusion in the choices of their treatment through active participation, enabling them to develop responsibility for their self-care [10]. This integration of the subject with their rehabilitation promotes the possibility of self-reflection, prioritization, and application of problem solving skills related to performance issues in the domains of activity and participation, developing self-care and autonomy [2, 11].

This new approach of physical therapy could guide the individual towards the empowerment of their own health, establishing the promotion of an intrinsic motivation by encouraging behavioral change [12] and thus, could increase the adherence to treatment and decrease the need for constant professional supervision for one to remain active. However, there are no intervention protocols that currently integrate conventional physical therapy (CPh) with this new approach of quality of care that includes the individual in the clinical decision to motivate and develop an empowerment of their own health.

Based on all information above, this study presents the EMPOWER-PD protocol, a physical therapy approach based on the individual preferences. As a new intervention protocol, it is necessary to verify it feasibility when applied to the target population. This study presents the design of the feasibility randomized controlled trial that aims to

(i) Investigate the feasibility of the EMPOWER-PD protocol through adherence rate, recruitment rate, and safety.
(ii) Compare the preliminary effectiveness of the EMPOWER-PD with the conventional physical therapy through qualitative analysis of motivation, empowerment of health, followed by the analysis of the dependency of professional supervision by individuals to remain active in the post follow-up period.
(iii) Compare the preliminary effectiveness of the EMPOWER-PD in motor and non-motor symptoms (functional mobility, gait, fatigue, aerobic capacity/endurance) with the conventional physical therapy through quantitative analysis of estimates of 95% confident interval.

We hypothesize that the EMPOWER-PD will lead to higher adherence and safety when compared with conventional physical therapy. Concerning motivation, health empowerment and dependency of professional supervision by individuals to remain active in the post follow-up period, we hypothesize that the EMPOWER-PD will be associated with better outcomes in preliminary effectiveness than the conventional physical therapy. For the motor and non-motor symptoms, we hypothesize that the EMPOWER-PD will be associated with similar preliminary effectiveness to the conventional physical therapy.

## Methods

### Idealization and development of the EMPOWER-PD

The idealization of EMPOWER-PD arose from the observation of the reality of the PD individuals and physiotherapists of the Brazilian Parkinson’s disease Rehabilitation Initiative (BPaRkI) (Florianópolis/Brazil). When the BPaRkI participants would return from a recess of activities (about 2 months), motor symptoms were always found to be worse than before. However, they would improve during the course of the treatment. We hypothesized that this was not purely the natural progression of the disease, but the physical inactivity and the lack of self-care in the absence of professional supervision. From these observations, three major facts were pointed out: the first is how the individual’s dependence on the professional delays their rehabilitation process; and the second is how we as professionals were corroborating for the increase and maintenance of this dependency. Another fact is that most of the participants of the BPaRkI came by medical recommendation with the diagnosis of the disease. This could explain in part their passivity towards their state of health and the reason why they are there.

Therefore, we began the search for a reality where the therapist succeeds in helping the subjects to help themselves. The concept of the centralization of the individual, as described by the gold standard guide of physical therapy in PD [2], was the central focus of the EMPOWER-PD. The purpose of this protocol is not the mere reproduction of the exercises in different individuals with PD, but rather the adaptation of the protocol to the actual needs and preferences of different groups of individuals. With this in mind, as a first step, we started listening to the BPaRkI participants. We created focus groups to identify preferences in leisure, expectations, exercise practice, pleasure, motivation, and daily routine. This can be considered as the most important step of the protocol, as it is very important to stimulate active participation in their state of health [9, 10, 13] and increase motivation.

Adding this information with the literature about the proven benefits of the physical therapy in PD [2, 3, 14–19], we started the development of EMPOWER-PD based on a mobility and locomotion training, as gait is considered one of the most potent indicators of disability in PD [20–22]. The protocol aims to provide a source of motivation and empowerment guiding the individual along this path through four goals. The first is the self-knowledge (first and second weeks) of their body through self-monitoring and self-management [2, 5, 8, 11, 23], where the individual reconnects body and mind with self-care [24]. In the second goal (third and fourth weeks), the individual approaches motor strategies during daily activities and during challenging exercises/situations. From this, the individual improves their ability to exercise [11, 25]. The third goal (fifth and sixth weeks) is focused on the stimulation of courage and confidence in their perceptions and convictions of how far they can care of themselves respecting their limits and their safety [23, 25]. In the fourth goal (seventh and eighth weeks), “the therapist moves away,” and the individuals choose their own exercises based on their preferences and on what they have achieved in the earlier weeks [5, 26]. The fourth goal seeks to provide an experience of independent self-care doing activities that are pleasurable and with people who have gone through the same process and that are equal to them regardless of the stage of the disease [2, 10, 26]. It should be noted that during all goals of intervention, the individual chooses the exercise intensity. For more details, see Table 1.

**Table 1:** EMPOWER-PD. 8-week physical therapy protocol, twice a week

Weeks/goals	Objective	Exercise	Instructions	Progression
First–second Self-knowledge	Stimulate self-knowledge: how does my body behave in the task?	From sitting, get up, and walk in an 8-m track, make the turn to the “U” (1 m), and walk diagonally to sit on the next chair.	Attentional focus during the task: pay attention to how you lift and sit, to the length and width of the pitch, arm swing and trunk movement, and their breathing, respectively.	The “U” will decrease (1 m–80 cm pivot) as well as the height of the chair (50 cm–45 cm–40 cm). Self-controlled speed.
Third–fourth Developing strategies for limitations and improving skills	Develop own strategies to overcome limitations (internal cues, imagery, sound stimuli, body adjustments). Enhance skills through their preferences.	From sitting, get up and walk in an 8-m track (passing and skirting obstacles, passing through narrow spaces (60 cm), holding objects) to the “U” (80 cm), interact with their preference, turn around, and sit on the next chair.	Attentional focus during the task: pay attention to how you go through the challenges. Realize where you have easy and difficulty. Test with your body the ways for you to overcome the difficulty and enhance what you have easily.	The “U” will decrease (80 cm–60 cm pivot) and the height of the chair (45 cm–40 cm bench). Obstacles will grow taller and narrower; the spaces will rise to 50 cm, and they will exchange objects between them during the gait.
Fifth–sixth Flexibilizing and developing confidence	Develop confidence and new strategies during walking and mobility in conflicting situations of daily living.	From sitting, get up, and walk 8 m under the same challenges of the previous week, but under more conflicting stimuli (talk to the duo during the task, different speeds, stop abruptly, traffic signal, sing, perform their concomitant preferences the task).	Attentional focus during the task: pay attention to how your body behaves in these conflicting situations. Test the strategies you already know and realize how safe you are by doing the task. Keep challenging yourself while always prioritizing your safety.	Pivot in all the ends and the height of the chair will continue of 45 cm–40 cm bench.
Seventh–eighth Winning autonomy and power for an active life	Decrease dependence on the therapist. Develop your own therapy based on your choices.	Booklet with the image of the circuit available for the free will to choose their exercises, their intensity, participants per season, and moment of progression.	As a team, choose how you guys will set up your circuit. I will be here to help you and listen to your doubts. Remember to respect your body and safety, but always progress using your individual strategies. Have fun!	At this stage, the therapist will be co-adjuvant in therapy, always stimulating the confidence and tranquility of the group and appearing in a few moments.

Legend: *m* meters; *cm* centimeters

One of the peculiarities of the EMPOWER-PD is the possibility to expand the learning with other participants in group. We believe that this is a very special aspect of this protocol because it provides benefits to both activity and participation [27]. As previously mentioned, the EMPOWER-PD comes as a new approach for individuals with PD and for the physiotherapists involved in this process. During the development of this protocol, we also seek to a new way of acting as a therapist. In this case, there could not be space for hierarchy and differences [7, 9, 12], since the re-encounter with health is in all people involved. As knowledge is shared either through professional expertise or through the experience of living with PD, it must be kept in mind that we are all “people” behind the role of therapist and patient and this is also what EMPOWER-PD seeks to put into focus.

### Feasibility and preliminary effectiveness design of the EMPOWER-PD

#### Study design and settings

**Fig. 1:**
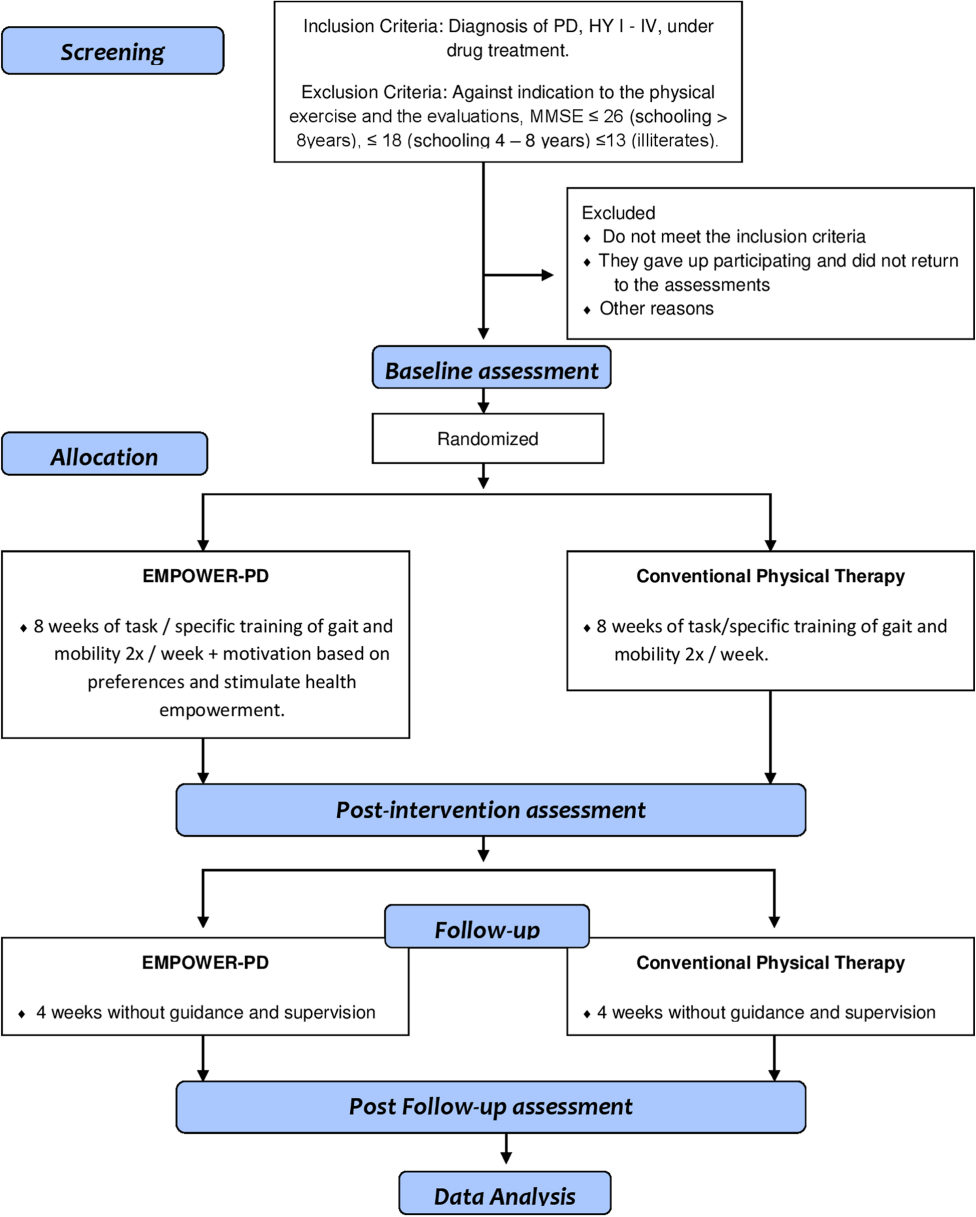
EMPOWER-PD feasibility study CONSORT-style flowchart

This 12-week pilot for a randomized clinical trial will assess the feasibility and preliminary effectiveness of the EMPOWER-PD and make comparisons with conventional physical therapy (CPh). The EMPOWER-PD intervention protocol was developed according to template for intervention description and replication (TIDieR) checklist [28] (see Additional file 1). The study protocol was described according to the SPIRIT guideline [29] (SPIRIT Checklist see Additional file 2), and its feasibility will be analyzed according to the aspects mentioned in the CONSORT checklist for pilot and feasibility clinical trials [30] (see Fig. 1). The research project was submitted to the University Research Ethical Review Board of the Santa Catarina State University (UDESC), Florianópolis, Santa Catarina, Brazil, according to the terms of Resolution 466/2012, and approved under report n° 2.145.010. The trial was registered on ReBEC–Brazilian Registry of Clinical Trials: Rio de Janeiro (RJ): Scientific Information and Technologic in Health Institute (Brazil); Registry name: A Physiotherapy protocol based on the preferences of the individual with Parkinson Disease for health empowerment, Identifier RBR-7ZBXQ5, Registered 18 August 2017, http://www.ensaiosclinicos.gov.br/rg/RBR-7zbxq5/.

Participants will be recruited from the BPaRkI of the Center for Health and Exercise Science of Santa Catarina State University–UDESC and written informed consent will be obtained from each participant before the initiation of any treatment. It is important to highlight that at any time; the participant may withdraw from the study without any prejudice. Data collection will be in clinical setting at Catarinense Rehabilitation Center in the city of Florianópolis/Brazil.

#### Sample size

The primary outcome of this pilot for a randomized clinical trial will be the feasibility (adherence, recruitment rates and safety). As in feasibility studies a formal sample size calculation is not required [30], we estimated the number of participants based on qualitative analysis. We will adopt the recruitment of 6–12 participants per group as a recommended for qualitative studies, being adopted the major value (*n* = 12 per group) [31]. Accordingly, we will use the criteria of sampling saturation that means “collecting data until no new information is obtained” [32].

In relation to the quantitative variables, the present study is not intended to be fully powered for detection of statistically significant effects, but it will enable a sample size calculation related to the quantitative variables for a future randomized trial [33]. For that, we plan to select the sample size based on reasonable precision of estimates of standard deviation of quantitative measures which have shown sensitivity in this feasibility study.

#### Randomization

A computerized block randomization procedure will be implemented by a blind researcher. Individuals with PD will be randomly allocated to two arms: (a) EMPOWER-PD or (b) conventional physical therapy (CPh). An independent person will notify the treating physiotherapists by email to ensure concealed allocation. The distribution of the groups will be kept in a closed envelope. The allocation will be on an equal ratio (1:1) between the two groups of interventions, stratifying by sex and by the stages I–III of Hoehn and Yahr scale (HY) [34]. To avoid bias, blinded testers will assess the participants.

#### Participants

##### Inclusion criteria

The inclusion criteria for recruitment will be participants with a diagnosis of Parkinson’s disease confirmed by the neurologist according to the UK PD Brain Bank Diagnostic Criteria [35], classified between the Stages of I to III according to the HY scale and stable medication regimen for at least 4 weeks before the intervention.

##### Exclusion criteria

Subjects with severe cognitive alterations that will impede the comprehension of questionnaires (score ≤ 26 for schooling over 8 years, ≤ 18 for schooling between 4 and 8 years, and ≤ 13 for illiterate participants in the Mini-Mental State Examination (MMSE)) [36], or those with contraindications for the practice of physical exercises will be excluded.

#### Interventions groups

The treatment sessions will be performed in group, twice a week, for 8 weeks, completing 16 sessions followed by a 4-week follow-up period. Before and after each session, blood pressure will be measured. The exercise intensity will be monitored every 5 min by an oximeter and the modified Borg scale [37] will be applied to verify the perception of effort in both groups by two physical educators for group. However, in the CPh group, the exercise intensity will be controlled between 55% and 85% of maximum heart rate (HR_max_) [2], according to the Karvonen’s formula. Each individual value will be calculated previously the interventions and vebaly stimulated during the session by the physical educator to keep up with the recommended HR values, increasing or slow walking speed. It is important to note that the intensity of the EMPOWER-PD protocol will be self-controlled according to the self-perception of effort of each one. The activities will be carried out during the “on” phase of the medication lasting 1 h, divided into 10 min of heating, 40 min of main part, and 10 min of back to calm [38] in an outpatient space.

##### Empower-PD

Before the intervention sessions, the physiotherapist responsible for applying the interventions will collect the preferences of each participant from an anamnesis with questions such as what is your routine on a weekday? and on the weekend?; what activity do you most enjoy doing in your routine?; what do you like to do in your free time?; do you practice any kind of art?; in what daily life activity can you perceive your body more?; and others if therapist judge necessary in their reality. With this information, the therapist can start to plan how to adapt the protocol to the participants, for example with music, materials used in leisure activities, group dynamics, musical instruments, materials used in their routine, among others.

**Fig. 2:**
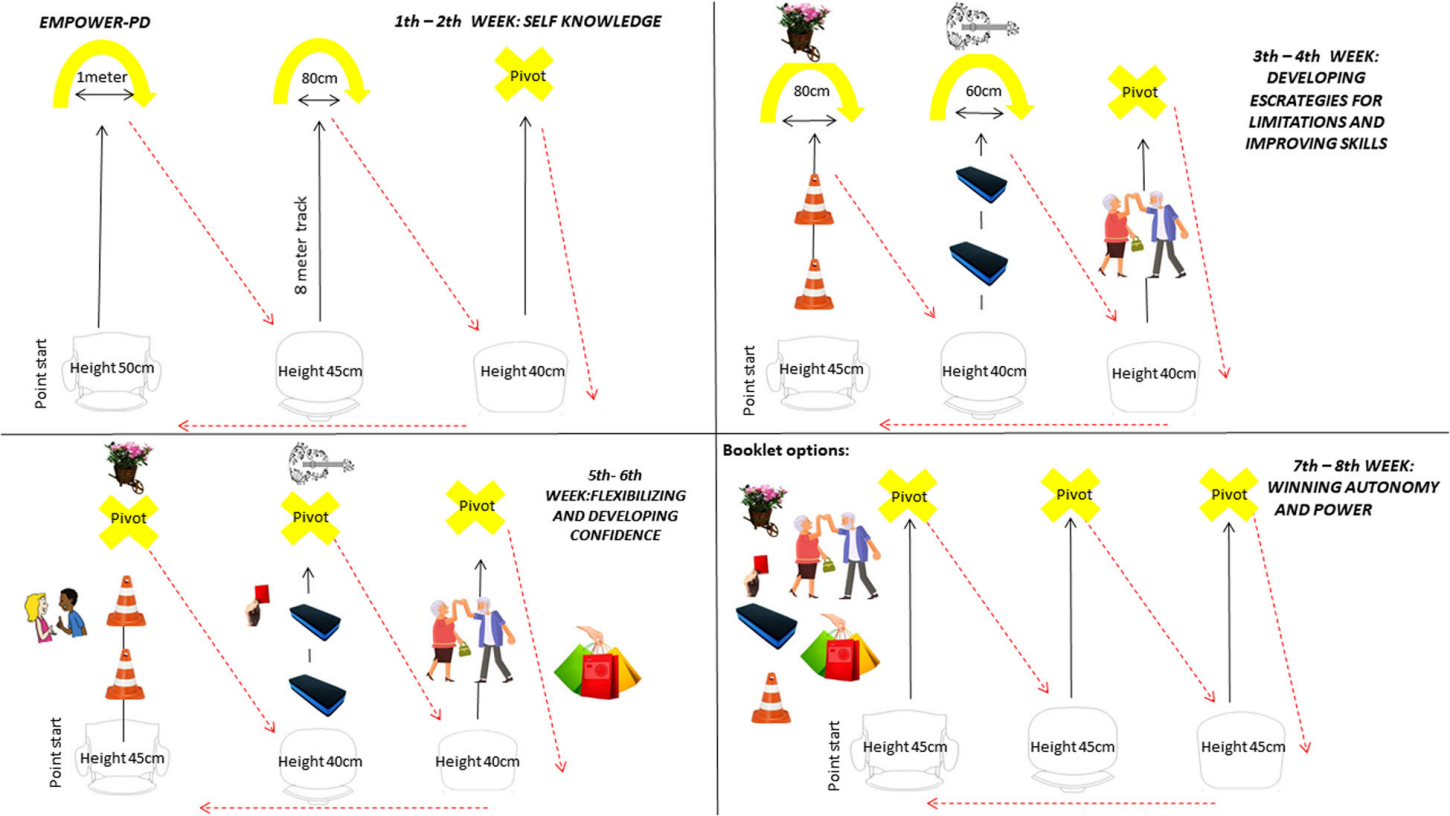
EMPOWER-PD protocol

All the participants recruited and enrolled to EMPOWER-PD protocol will be training in a mixed circuit, at the same time, involving walking in solo training in different contexts (crossing obstacles, reaching activities, circumventing obstacles, passing through narrow spaces, dual-functional-task walking) (Fig. 2). In addition, to sit and stand training and the pivot with progressions will be specific to each task. It will be applied from the main part of 40 min duration, being allowed pauses to rest according to the individual’s perception. The main part starts with all participants inside the circuit. Some individuals will be sitting and in the other side, the others will be standing. When the therapist or the participant said to start, all will move together to the next station. Each station will require different tasks that will progress every 2 weeks focused on their activities of daily living and in challenging exercises according to motor control principles. The circuits will include mounted steps, chairs at different heights and benches, cones, red, green and yellow signs, items of preferential use (gardening, musical instruments, cleaning), which are behind the “U” or pivot-floor marking. The interaction with the preferred items will be done when the participants are in the “U” or pivot, during the period that the whole group went to their positions. Detailed descriptions of the interventions and their progressions are presented in Table 1.

Every 2 weeks of intervention, one pillar of EMPOWER-PD will be addressed (self-knowledge of your body (first), developing strategies for your limitations and enhancement of your abilities (second), flexibilization and development of your abilities (third) and autonomy and empowerment of health of participants for a more proactive life (fourth)). The two first weeks are periods of familiarization with the dynamics of the exercise and with the new stimulus of self-perception (for more details see Table 1). From the third week on, several materials will be introduced according to the protocol. All participants will be entering in contact with the preferences of all in a circuit system, where each step the individual will interact with a different activity and object of preference, for example, dance, sing, paint, cooking, gardening, and others, according to group and therapist creativity. The therapist will assume the role of group guide, in front of the exercises and always generate positive feedback to each exercise instruction. The positive feedback provided will be done verbally after each task, with positive reinforcement and incentive phrases during the practice of the exercise.

Specifically, in the last 2 weeks of the protocol (seventh and eighth weeks), the fourth pillar of EMPOWER-PD (winning autonomy and power for an active life), will be composed by a booklet (Additional file 3) with circuit images and suggestions of dynamics and progressions (already done in weeks 1 to 6 of the protocol) that will be chosen by the participant based on their preferences. The booklet will be formulated by the therapist according to the information from the group collected by contact with them in all sessions, being adapted for each population that receive the EMPOWER-PD. It will be composed of multiple choice options of stretching, main part, progression, preferences, and cooling; all chosen according to the group previously at the beginning of the activity. Throughout the sessions of the last 2 weeks, the therapist will become less active in the stages of construction of the exercises, duration, and frequency of the exercise, passing such responsibility to the individuals themselves. The intervention will continue to be supervised, but the therapist will be present as a source of guidance stimuli for the group, how initially controlling the time of each part, suggesting the time to progress and others according to the necessity of the group.

The warm-ups will always count on varied exercises that stimulate the activation of the body’s own perception and the relationship between the groups. The exercises will use music, interaction with their own body and their colleagues, and their suggestions of warm-up exercises based on their own knowledge about physical activity previously practiced. A “back to calm” stage will feature breathing and meditative exercises with the goal of to perceive sensations in their body after the exercise. In addition to the feedback provided after each task, at the end of each session, a talk wheel is held about their perceptions of the session and the repercussion under their daily routine.

##### Conventional physical therapy (CPh)

**Fig. 3:**
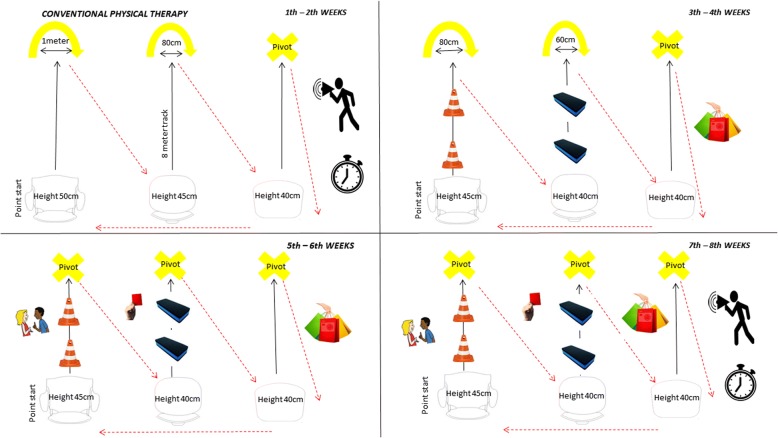
Conventional physical therapy protocol

The sessions are given by two physiotherapists and unlike EMPOWER-PD, a conventional physical therapy will not address motivational factors available in the preferences of the individual and during all sessions. The physiotherapist will be the main responsible for assembling, controlling, and directing the interventions in relation to each proposed exercise, their durations, intensities, and frequency for all the 8 weeks (Fig. 3).

The therapists will have free choice to use feedbacks, and their instructions will be more related to the execution of the exercise than to the stimulus of self-knowledge of the patient. In this way, the warm-ups will have active joint mobilization exercises, increasing the speed of movement progressively according to the therapist’s command to the group. In the “back to calm” stage, breathing exercises will be performed, starting from diaphragmatic exercises to sustained inspiration and inspiration in times with the association of the upper limbs.

The training will follow the same EMPOWER-PD base structure (mixed, varied, and in blocks), featuring walking in solo, sit-and-lift, and pivot training with specific progressions for each stage of the circuit and increasing the level of difficulty of the complete task by increasing the speed from the therapist’s command. All the participants recruited and enrolled to the CPh will be training at the same time as well as in the Empower-PD group. However, we will control the exercise intensity (55 to 85% of HR_max_, as described previously). The therapy will be in group but without doubles or trios. The materials used in this protocol are the same as the EMPOWER-PD with the addition of the timer to control the execution speed of the circuit in some stages of the CPh protocol. The description of the exercises and their progressions during each week are explained in Table 2.

**Table 2:** Conventional physical therapy. 8-week physical therapy protocol, twice a week

Weeks/goals	Objective	Exercises	Instructions	Progression
First–second	Increase speed of execution from a comfortable speed to a fast speed.	From sitting, get up and walk in an 8-m track, make the turn to the “U” (1 m), and walk diagonally to sit on the next chair.	Get up from the chair and walk to the “U,” go around it and sit on the next chair. Start by performing at comfortable speed, with each command increasing speed (“a little faster”/“fast”/“as fast as you can without running”).	The “U” will decrease (1 m–80 cm pivot), and the height of the chair (50 cm–45 cm–40 cm). Every three repetitions of the circuit will be given the command to increase the speed.
Third–fourth	Develop the ability to adapt gait and sit and lift to different sensory contexts.	From sitting, get up and walk 8 m (passing and skirting obstacles, through narrow spaces of 60 cm, holding objects and etc.) to the “U” (80 cm), skirt and sit on the next chair.	Get up, walk, go through the challenges encountered along the circuit until you reach the “U,” go around it, and return to sit on the next chair.	The “U” will decrease (80 cm–60 cm pivot) and the height of the chair (45 cm–40 cm bench). Obstacles will grow taller and narrower; the spaces will rise to 50 cm, and they will exchange objects between them during the gait.
Fifth–sixth	Develop the ability to adapt gait and sit and lift to different sensory contexts under conflicting situations of double task.	From sitting, get up and walk 8 m under the same challenges of the previous week, but under more conflicting stimuli (talk during the task, different speeds, stop abruptly, traffic signal).	Get up, walk, go through the challenges encountered along the circuit until you reach the “U,” go around it, and sit on the next chair. I will post different conflicting situations in the course of the exercise.	Pivot at all ends and the height of the chair will continue from 45 cm–40 cm bench. The progression will be made in the following order: noise in the room, talk during the task, different speeds, stop abruptly, and traffic signal.
Seventh–eighth	Increase running speed of the walking circuit and sit and lift in different sensory contexts and conflicting situations (≤ 3 s from first to last lap).	From sitting, get up and walk by 8 m under the same challenges of the previous week, but under more conflicting stimuli (talk during the task, different speeds, stop abruptly, traffic signal).	Get up, walk, go through the challenges of the circuit until you reach the “U,” go around it, and sit on the next chair. I will post different conflicting situations during the exercise and every three laps I will ask you to increase the speed (“a little faster”/“fast”/“as fast as you can without running”).	The progression will continue the same as the previous week, but the increase speed command will be added every three repetitions of the circuit.

Legend: *m* meters; *cm* centimeter

#### Outcomes

##### Primary outcomes

**Fig. 4:**
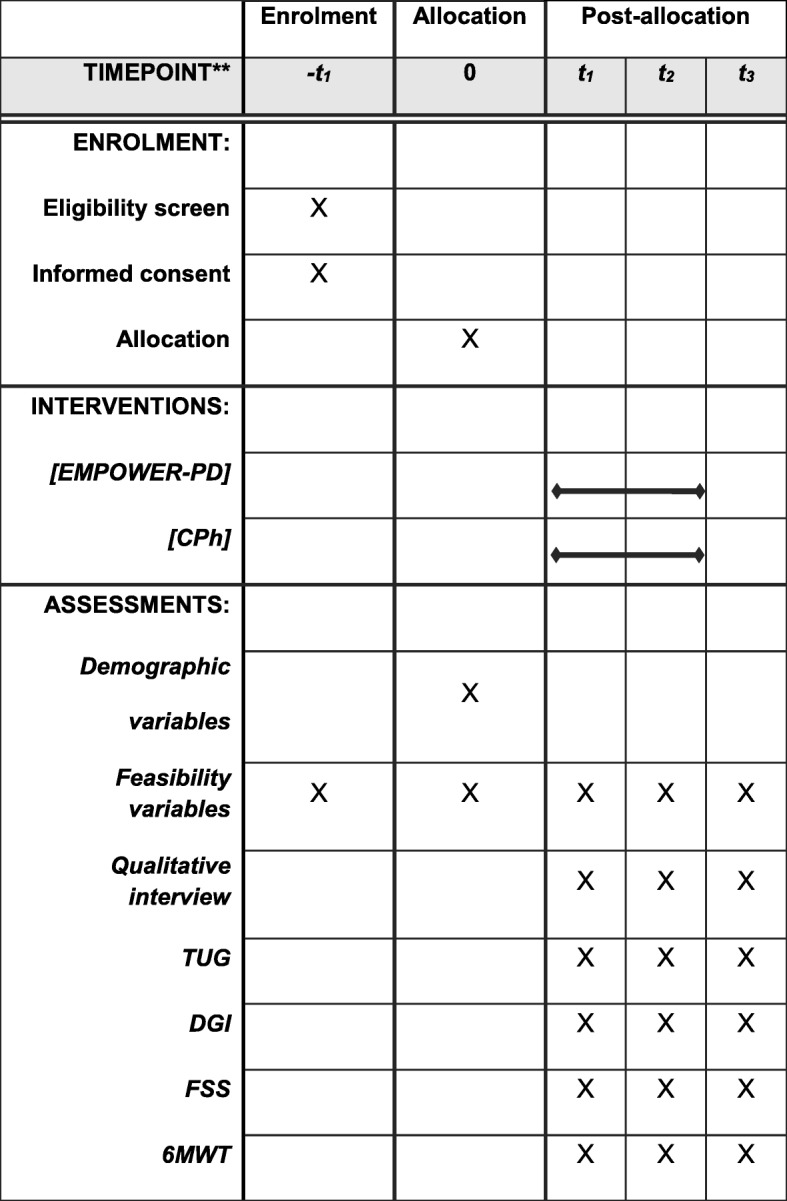
Schedule of enrolment, interventions, and assessments. SPIRIT recommendation. Legend: t-1, before allocation; t0, allocated; t1, baseline; t2, post-intervention; t3, post-follow-up; CPh, conventional physical therapy; TUG, Timed Up and Go; DGI, Dynamic Gait Index; FSS, Fatigue Severity Scale; 6MWT, 6-min walk test

Adherence will be assessed through retention and attendance rates. The retention rate will be analyzed by the percentage of participants completing all sessions of the intervention (16 in total) as well as the percentage of participants present in each step of study until the last evaluation at the follow-up period. Attendance rate will be analyzed by the mean of presences of the participants divided by the number of sessions offered during the 8-week intervention, multiplied by 100. The recruitment rate will be expressed by percentage and analyzed by the number of eligible individuals recruited per month. We will consider a recruitment and retained rate of > 80% as feasible to conduct a future randomized control trial. Safety will be reported by the number of adverse events. Adverse events were defined as exercise intolerance, injuries, or falls during the intervention [39], considering < 5% of occurrence of adverse as acceptable to conduct a future randomized control trial. The acceptability of the protocol by the participants will be better elucidated by the qualitative analysis in a general way (Fig. 4).

##### Secondary outcomes

The qualitative assessment about motivation, health empowerment, and the dependency of professional supervision at post follow-up period will be done by two researchers during an in-person interview, in a private room, and recorded for further transcription line-by-line and analysis. The questions will follow investigating the motivation in different steps of the study and are described in Table 3:

**Table 3:** Distribution of questions in the study steps

Steps	Week	Question
Baseline	0	1. What made you seek physical therapy? 2. What does Parkinson’s disease mean in your life? 3. What are your expectations with physiotherapy?
Post-intervention	8	4. What this physiotherapy program meant in your life? 5. What did you think about the physiotherapy program that you participated? 6. After physical therapy, do you think you will be able to perform the exercises at home after discharge? What made you feel more or less capable? 7. Now at the end of the supervised treatment, what is your opinion of the expectations that you had at the outset?
Post follow-up	12	8. Were you able to do this exercise without supervision in your daily life? a) How was your experience? b) If you cannot do, what were the reasons? 9. After this period without supervision, what has this physiotherapy program meant in the last months of your life? 10. After your participation in this study, the reasons that make you seek physiotherapy are the same?

Note: Script assembled after pretest and familiarization with three individuals with PD.

The quantitative outcomes will investigate the preliminary effectiveness of the EMPOWER-PD on mobility, gait, fatigue, and aerobic function/endurance. Mobility will be assessed by the Timed up and Go test (TUG) which consists in walk a distance of 3 m, go beyond the horizontal mark located on the floor, turn on its axis, and return to sit again on the same chair. The time of all tasks will be timed and classified according to falls risk for PD individuals (< 11 s) [40, 41].

Gait will be evaluated by the Dynamic Gait Index (DGI) that assesses the gait in different sensorial contexts for eight tasks, which will be classified in a score of 0 to 3. The maximum score is the 24 points indicating a gait performance capable of adapting the different demands proposed by the test [42].

To assess the fatigue, we will use the Fatigue Severity Scale (FSS). The scale evaluates the impact of fatigue on motivation, exercise, physical functioning, accomplishing tasks and responsibilities, and interfering with work, family, or social life. Contains 9 items, in which the rating scores range from 1 to 7 for each statement. The highest scores (maximum 63 points) indicate more severe fatigue [43].

The 6-min-walk test (6MWT) will quantify the aerobic function/endurance. The participants will walk 30 m for 6 min, spinning the cones positioned in the final 50 cm after the starting and finishing point of the track. The total number of laps is multiplied by 60 (30 m + 30 m) and added to the distance in meters from the last lap resulting in the total distance covered in the test [44, 45].

### Procedures

The baseline assessment will be conducted once participants have given informed consent. All interviews/scales will be applied individually in a private setting by a blinded researcher, and all participants will receive an identification number. All evaluation will be performed in the afternoon, with individuals on the “on” phase of the medication. Demographics and eligibility criteria information will be collected at the baseline step. From there, the volunteers eligible in the inclusion criteria will be randomized into one of the two groups (EMPOWER-PD group and conventional physical therapy group) by a second researcher not involved in other steps.

Participants allocated to both groups will receive 8 weeks of intervention that will occur at the same time in different places to minimize individuals’ awareness of the differences between interventions. Then, the participants will be assessed at post-intervention, always by the same physiotherapist research, followed by a follow-up period of 4 weeks. The main objective of this stage of the study is to verify if the intervention would be a source of motivation/empowerment of health for a more proactive life, and in this way, decrease the dependency of professional supervision to remain active. For this, the participant will remain 4 weeks without any guidance from the therapist. After this, they will be contacted by phone to schedule the final evaluation, which will be performed on all outcome variables of the others stages of the study. Participants who drop out of the study will also be contacted by phone for the final evaluation.

### Data management and analysis

For the feasibility analysis, the results will be expressed as percentage and numbers referring to adherence, recruitment rates, and safety, respectively. After qualitative data collection, two authors will perform the total transcription of the audios and notes line-by-line. Then, two authors will start the codification of the *corpus.* After transcription, the corpus will be codified in numbers and their respective groups (e.g., # ID1emp, # ID5cph) by a blinded third researcher, for the later analysis by a fourth researcher. Codes will then be consolidated into categories after an extensive exploration of the material and interpretation of the data obtained according to thematic analysis of the content. From this, entire research team will discuss to formulate inferences.

To verify the preliminary effectiveness regarding quantitative outcomes, the descriptive analysis will be performed with frequency, central tendency, and variability (mean and standard deviation or median and interquartile interval) in the statistical program SPSS 20.0 for Windows (IBM-USA). All results of the preliminary efficacy of the feasibility trial will be described as estimates of 95% confidence interval. An intention-to-treat analysis will be done with participants including who eventually will be missed in any part of the study.

## Discussion

It is widely mentioned in the literature that individual with PD have important barriers in adherence and motivation with regards to maintaining an active lifestyle [3, 5, 6]. However, few studies have sought practical changes to increase individual’s motivation and empowerment, most of which are qualitative [5, 11]. EMPOWER-PD is innovative because it has the potential to identify and harness intrinsic motivation [2, 12] (through the self-knowledge of its capacities and limitations), developing with the participant, a path to empowerment of their health. We believe that this kind of approach, centered on individual preferences, would provide a source of motivation/empowerment of health and perhaps decrease the dependency of professional supervision to remain active.

In this way, this study protocol was developed to analyze the individual in its totality, including their expectations and perceptions of their own body. Based on this, the design of the pilot study was planned, aiming to investigate the feasibility aspects such as adherence/recruitment rates and safety as well as the receptivity of the participants to this new physical therapy approach. The preliminary effectiveness regarding motor and non-motor symptoms will be also addressed generating future data for sample size calculations for an adequately powered analysis. In addition, from the qualitative variables, the pilot study will investigate the meaning of the protocol in their life, the real motives that prevent the continuity of physical exercise practice, and autonomy in the management of their health [5, 7]. Through interviews, individuals have the opportunity to verbalize their reflections on the perceived outcome of rehabilitation, leading to an awareness of the efficacy of an active life and their own limitations and capabilities, allowing the passage of time the patient’s passive state to a more proactive disposition [5, 9].

This protocol is the first step towards a new vision of physical therapy care for both PD individuals and therapists [7]. Its validation and application will bring many other issues, changes, and limitations that will come along the way, enabling your deepening, enhancement, and incentive by futures clinical trials. However, the message the EMPOWER-PD carries and what we want to find on this path is that the exercise is no longer a medical obligation, but an act of care with itself. This act of caring is the source of approximation of the people involved in this seeking to live empowered health and well-being with themselves and with the others. The present study hopes to stimulate many other studies, protocols, and revisions that aim at studying the patient-centered approach to empower PD individuals. Believing that the efficacy of specialized physiotherapy in PD is maintained [2, 3, 46], but with a different way to see the therapist and the individual with PD [2, 12]. If feasible, this pilot randomized controlled trial may help guide a larger, definitive, randomized controlled trial to determine the effectiveness of EMPOWER-PD intervention in patients with Parkinson’s disease.

### Trial status

The EMPOWER-PD trial started in December 2016. Recruitment commenced in August 2017 and will continue until March 2018. The end date for the trial is October 2018.

## Additional files

Additional file 1: Template for intervention description and replication (TIDieR) checklist of the study protocol. (DOCX 29 kb)

Additional file 2: Description of study protocol according to the SPIRIT Checklist. (DOC 122 kb)

Additional file 3: EMPOWER-PD (WINNING AUTONOMY AND POWER FOR AN ACTIVE LIFE) booklet. (DOCX 1743 kb)

## Abbreviations

BPaRkI: Brazilian Parkinson’s Disease Rehabilitation Initiative
CONSORT: Consolidated Standards of Reporting Trials
CPh: Conventional physical therapy
HR_max_: Maximum heart rate
HY: Hoehn and Yahr
ICF: International Classification of Functionality
MMSE: Mini-Mental State Examination
PD: Parkinson’s disease
SPIRIT: Standard protocol item
SPSS: Statistical Package for the Social Sciences
TIDieR: Template for intervention description and replication
TUG: Timed Up and Go
UDESC: Santa Catarina State University
UK: United Kingdom
