# Supplementary material for: EMPOWER-PD - A physical therapy intervention to empower the individuals with Parkinson’s disease: a study protocol for a feasibility randomized controlled trial
Source: Pilot Feasibility Stud. 2019 Jan 28;5:19. doi: 10.1186/s40814-019-0394-9 (PMC6348670; doi:10.1186/s40814-019-0394-9)
Supplement: Supplementary file 3 — EMPOWER-PD (WINNING AUTONOMY AND POWER FOR AN ACTIVE LIFE) booklet. (DOCX 1743 kb) [file 40814_2019_394_MOESM3_ESM.docx]

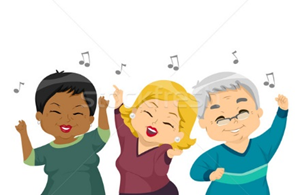

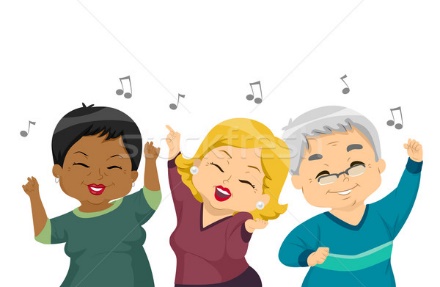


HOW MUCH AM I FEELING TIRED?

1. Nothing tired.
2. A little tired.
3. Very little.
4. Moderately.
5. A little had.
6. Hard.
7. Very hard.
8. Harder.
9. Extremely hard.
10. Maximum effort.
11. Fatigue.


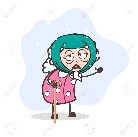


MENU FOR FUN RIDING OUR WORKUT

1.What I like to do? Mark (x)

-
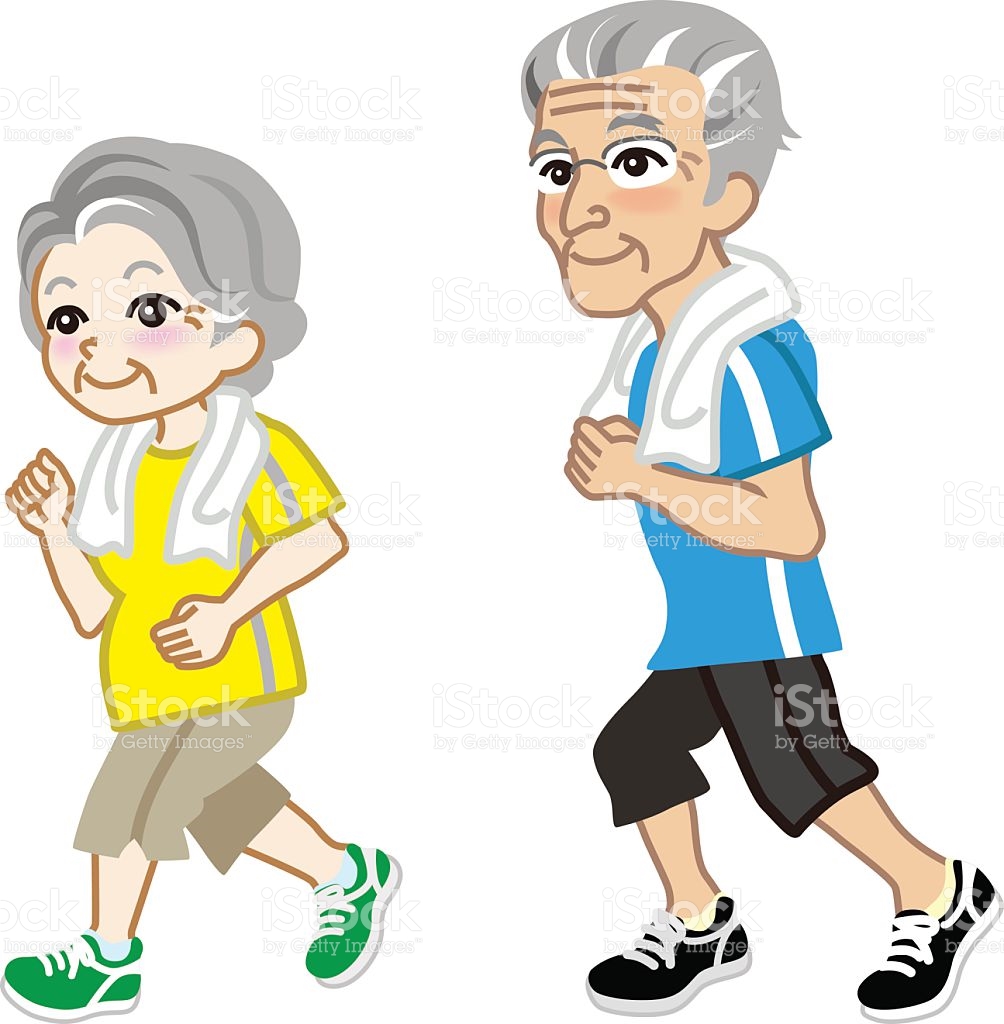
 To run.

OUR TIPS TO ROCK

- Transfer weight fast when you feel your foot.
- Imagine how perform the challenge before you run it.
- Give attention to our pains.
- Respire.
- Challenge yourself with security.
- When does the speed increase and when does it decelerate?
- More concentration on difficult tasks.

1

2

3

4

-
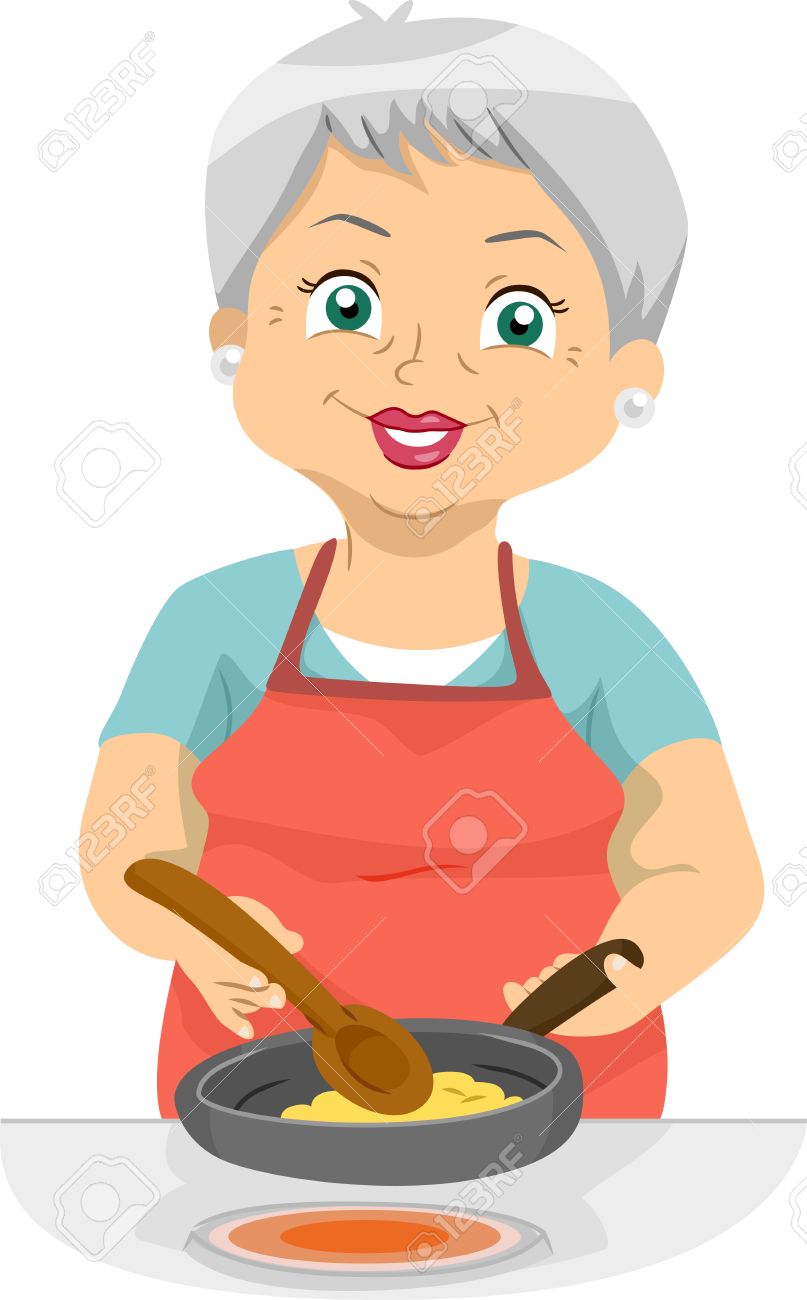
 To cook.
-
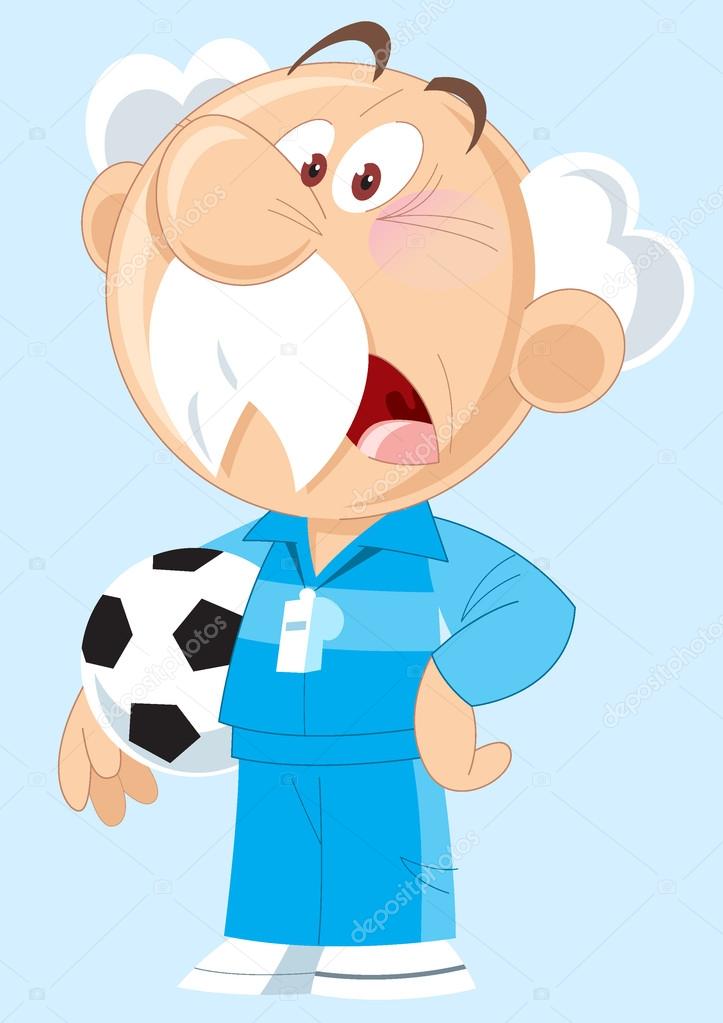
 To play soccer.
-
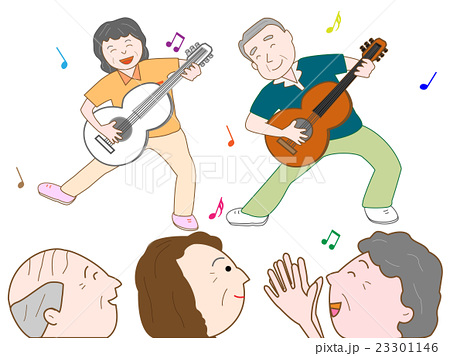
 To Sing.
-
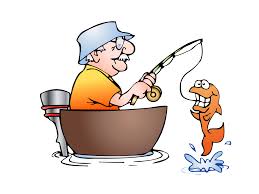
 To fish.
-
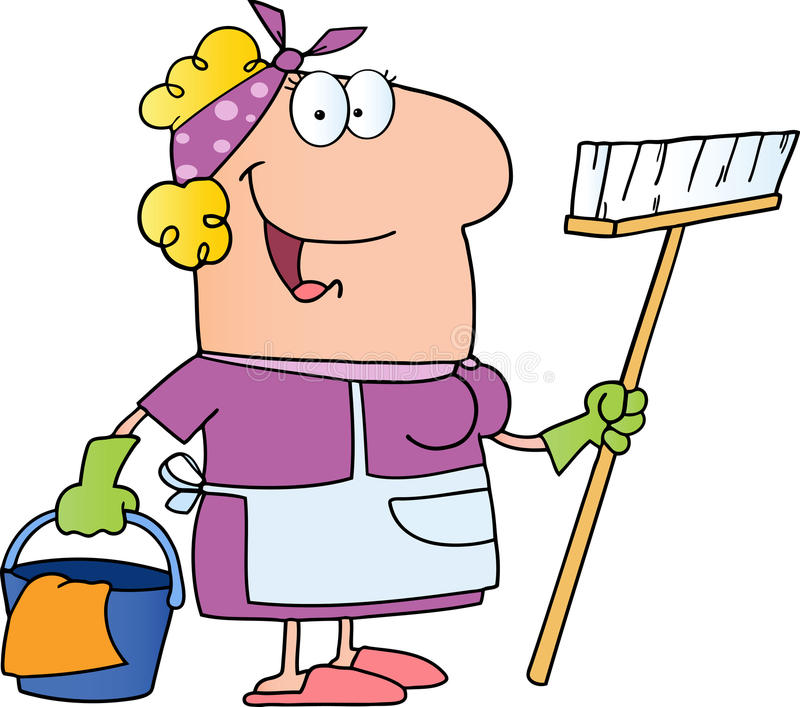
 To clean the house.
-
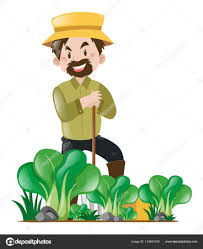
 To take care for garden.

TRAINING BOOKLET


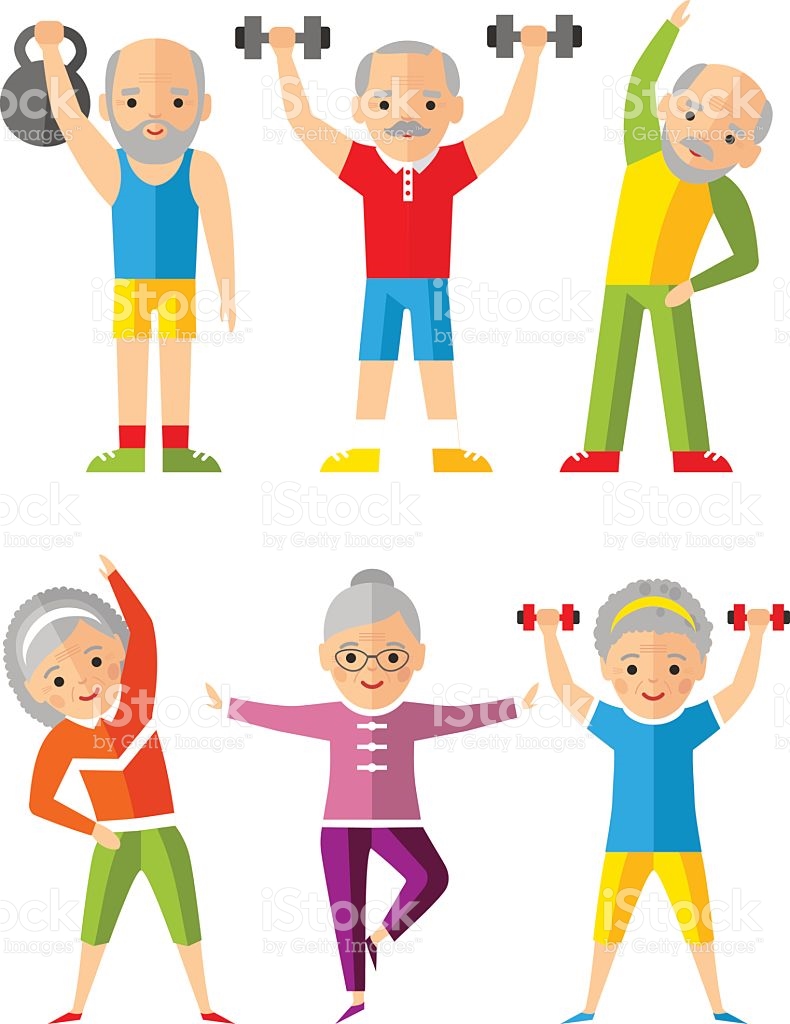


DIVISION

10-minute warm

10-minute exercise

10-minute go back to calm


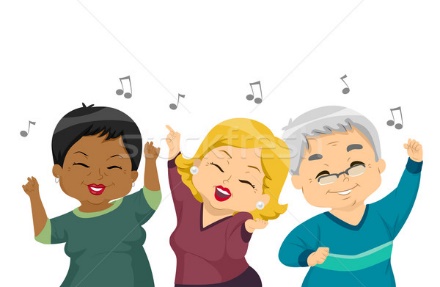


4. What is the intensity?

- Comfortable walk.
- Quick walk.
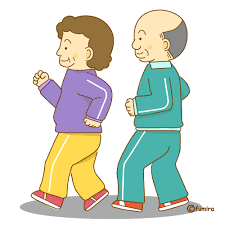


I´m challenging myself?

- I'm confident to challenge myself more;
- I go to respect my safety.

How tired am I?

- From 0 to 10.

c

6. GO BACK TO CALM

- Breathe;
- Feel the body, and;
- Reflect on what you felt.


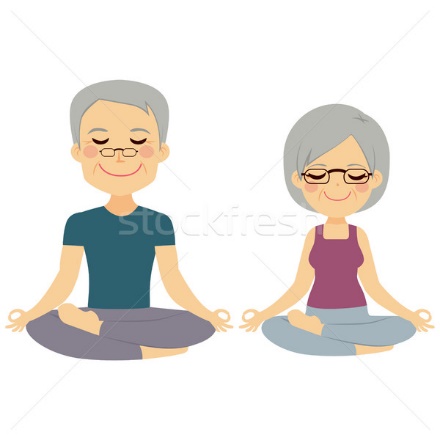


Is it easy for me, let's progress?

Physical challenge

- To cut obstacles;
- Pass through narrow spaces;
- Pick up and pass objects;
- Other.


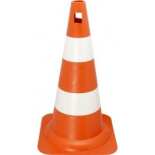

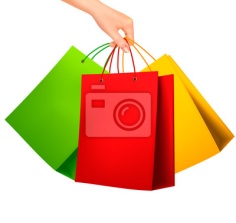


Challenge of the mind

- Talk and walk;
- Semaphore;
- Sing and walk;
- Walk fast, slow and stop.


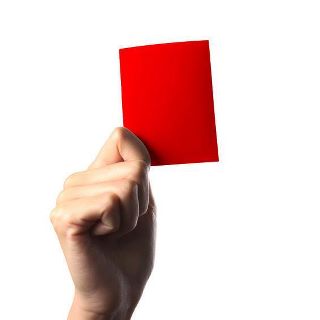

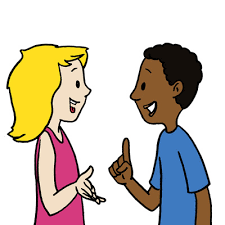


How is my breath?

- Gasping;
- Slightly breathless;
- Normal.


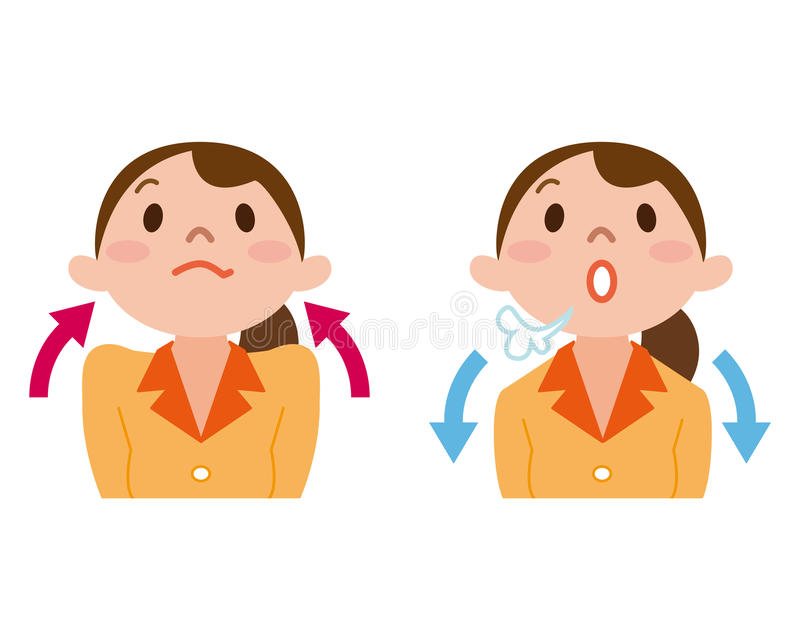


5. EXERCISE

Is it easy for me, let's progress?

Physical challenge

- To pass obstacles;
- To dance;
- To Walk.


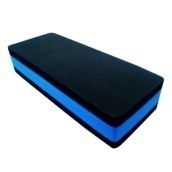


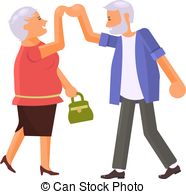

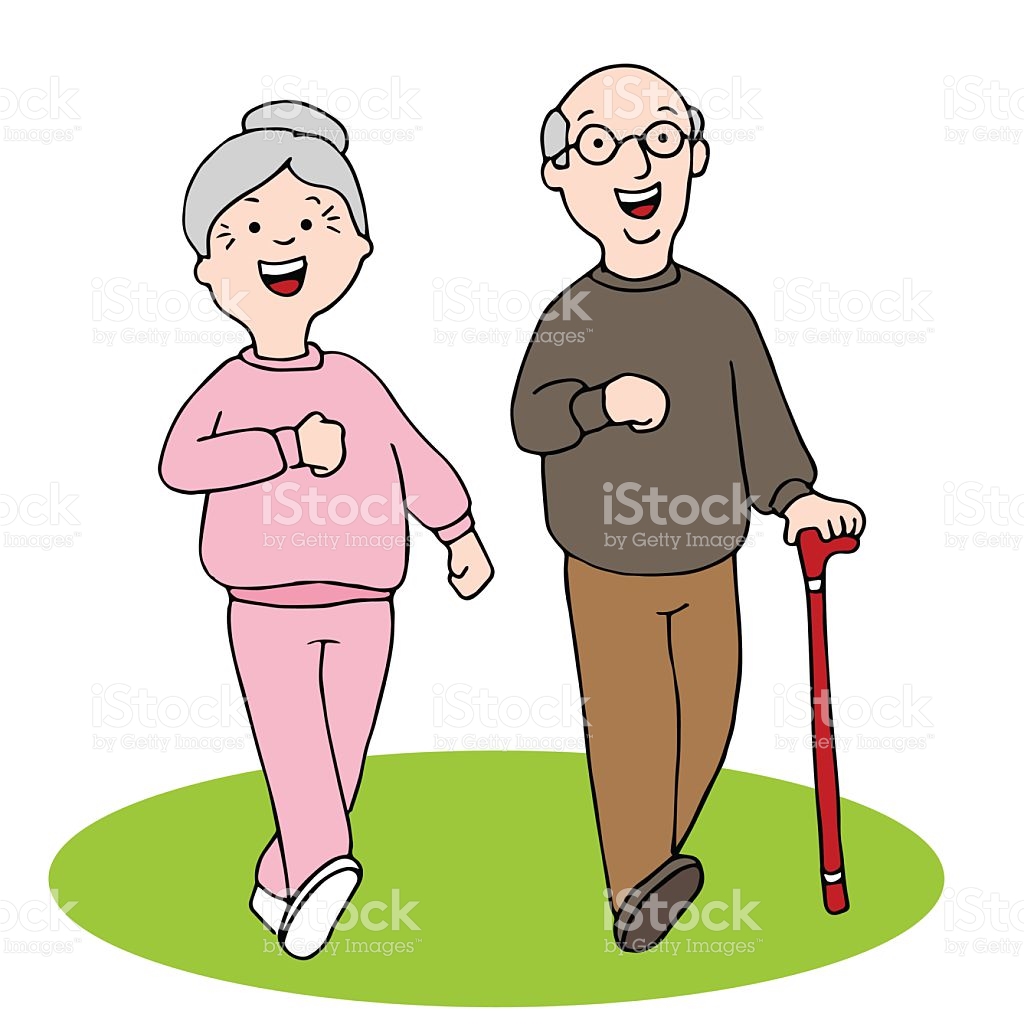


2.TASK DIVISION

- Triplet.
- Dual.
- Individual.

3.WARM

- Stretching (30 minute).
- To dance.
- To move the joints.


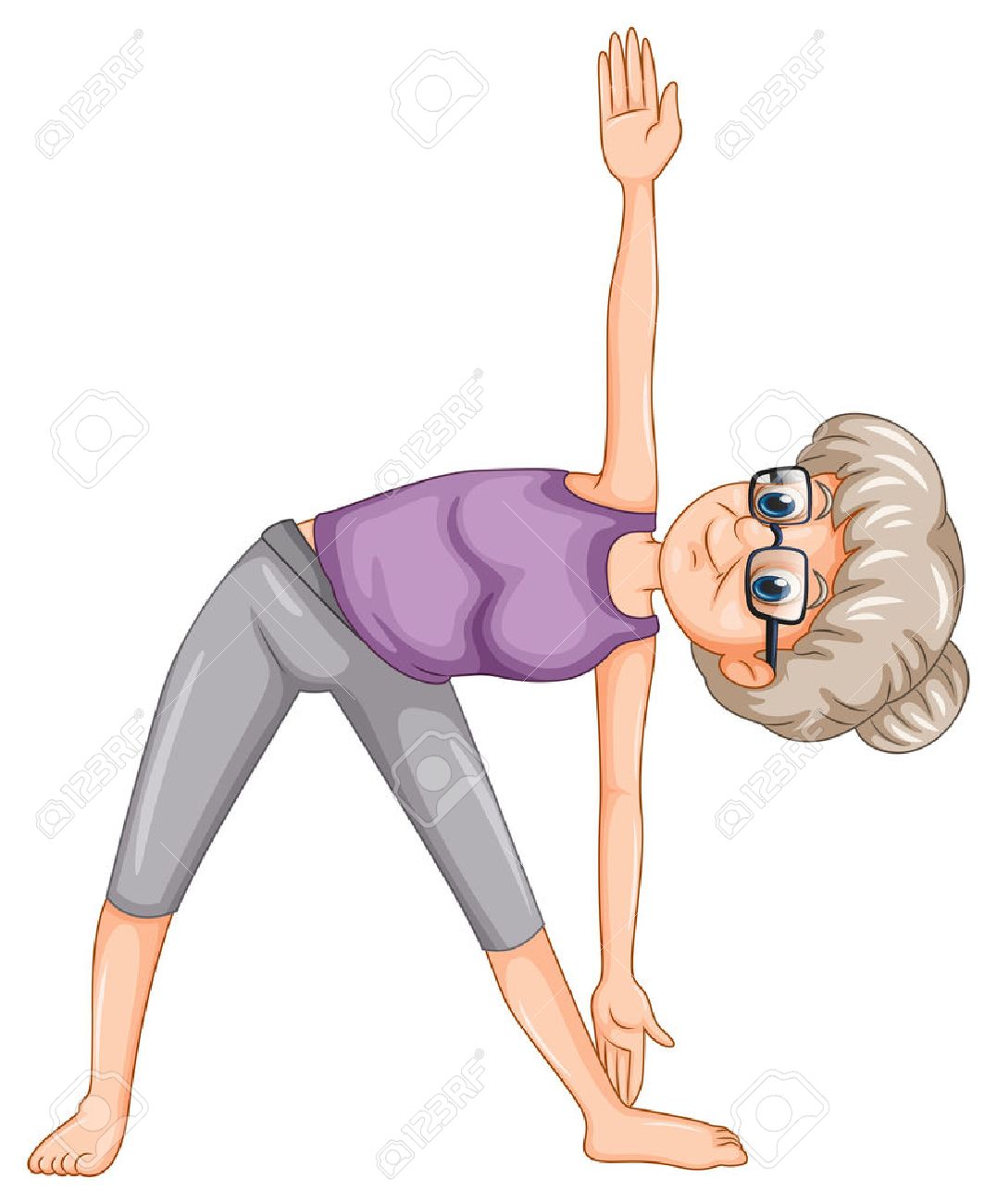

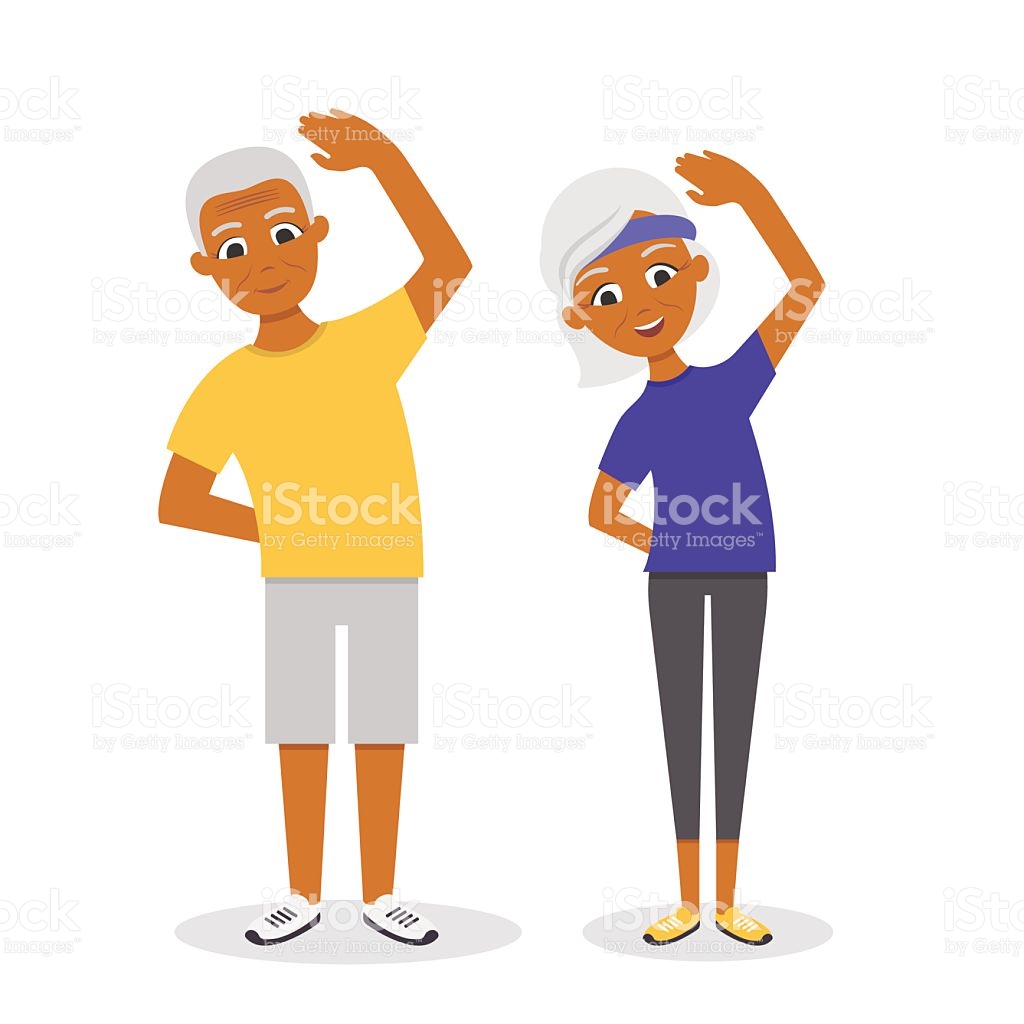


5

6

7
